# Personal News Items

**Published:** 1914-11

**Authors:** 


### Personal News Items.

Dr. and Mrs. Warren, of San Angelo, have located in Boerne, Texas.

Dr. Fourton, of Center Point, has returned from a trip to El Paso.

Dr. Callonder L. Johnson, of Dallas, has returned from a trip to the East.

Dr. M. Z. P. Blackstone, of Kemp, Texas, is soon to locate in Huntington.

Dr. and Mrs. J. P. Blount, of Denton, are home from Hot Springs, Ark.

Dr. J. B. Winn, of Hamilton, who has been quite ill, is very much improved.

Dr. S. T. Humphreys, of Nocona, Texas, recently visited Dallas and Fort Worth.

Dr. Guy Morgan, of Petty, has recently returned from a three months' trip abroad.

Dr. J. J. Morris, of Dallas, has returned from a three months' visit to Boston and New York.

Dr. J. M. Campbell, of Goldthwaite, has returned from Temple, where he spent several days in the sanitarium.

Dr. and Mrs. J. Saunders Cooper, of 2011 Bennett Avenue, Dallas, are rejoicing over the arrival of a little daughter.

Dr. John H. Barnett, formerly of De Leon, Texas, has located at China Springs, and is now ready to do general practice.

Dr. A. G. Shortle's article next month on "Artificial Pneumothorax" will be especially interesting.

Dr. J. V. Blake, of Floresville, has established his reputation as an angler, having recently caught a tarpon which measured six feet from tip to tip.

Dr. Charles M. McMillan, of Plantersville, has recently returned from Chicago, where he completed some very valuable courses in human anatomy.

Dr. Chas. H. McCollum, of Hamilton, who spent the past year taking special work in Austria and several other European countries, will locate in Fort Worth and do special work only.

Dr. and Mrs. David Lawrence and children, Miss Katherine and Master David, Jr., of Galveston, have returned from Fort Davis, where they spent the summer.

Dr. Chas. B. Buck and family have returned to Mercedes. Mrs. Buck and children have been visiting in Oakland, Kansas, for the past four months, and the doctor joined them there about a month ago.

Dr. R. H. Harrison, of Bryan, had the misfortune to fracture both bones in the right wrist while cranking an automobile. The arm was given medical attention as soon as possible, and is now getting along as well as could be expected.

Dr. A. W. Carnes, of Hutchins, had a very narrow escape recently. While driving his auto along the pike he reached for a package which was about to make its escape from the machine, and turned the steering wheel, running into a ditch, where it turned over, with the doctor underneath. He was held a prisoner until the constant tooting of his horn attracted someone who came to his rescue. He was fortunate in escaping with only slight bruises.

